# Phospholipid Networks as Metabolic Hubs and Signaling Integrators in Plant Development and Stress Adaptation

**DOI:** 10.3390/plants15091404

**Published:** 2026-05-04

**Authors:** Pengjie Chang, Ming Ju, Hengchun Cao, Yinghui Duan, Qiuzhen Tian, Cong Mu, Guiting Li, Xiaoxu Feng, Weixiu Hou, Haiyang Zhang, Hongmei Miao

**Affiliations:** 1 Henan Sesame Research Center, Henan Academy of Agricultural Sciences, Zhengzhou 450002, China; 2 Henan Key Laboratory of Specific Oilseed Crops Genomics, Henan Sesame Research Center, Henan Academy of Agricultural Sciences, Zhengzhou 450002, China; 3 Henan International Joint Laboratory of Specific Oilseed Crops Improvement, Zhengzhou 450002, China; 4 College of Agronomy, Henan Agricultural University, Zhengzhou 450046, China

**Keywords:** phospholipids, lipid signaling, auxin transport, phosphatidic acid, cellular reprogramming

## Abstract

Phospholipids function as dynamic regulators of plant growth and environmental adaptation, extending well beyond their structural roles in biological membranes. This review synthesizes the phospholipid metabolic network and its regulatory functions in plant physiology. We first describe enzymatic reactions and acyl-chain remodeling in phospholipid biosynthesis, and then examine the interaction between phospholipid metabolism and auxin signaling, focusing on phosphatidic acid (PA) and phosphoinositide phosphate (PIP). These lipid molecules regulate the polarization and vesicular trafficking of PIN-FORMED proteins via endocytosis and phosphorylation-dependent mechanisms, thereby controlling auxin distribution during development and stress adaptation. Particular emphasis is placed on PA, a multifunctional signaling lipid that serves as a central molecular hub. PA coordinates hormonal, stress, and circadian signals by engaging and modulating a broad spectrum of protein targets, including kinases, phosphatases, and transcription factors. We also discuss the emerging and evolutionarily conserved functions of phospholipid signaling in cell fate determination, drawing parallels from mammalian cell reprogramming to the regulation of plant cell totipotency and root patterning. Collectively, these findings underscore the critical role of phospholipid-mediated signaling in converting metabolic and environmental cues into developmental reprogramming, providing novel theoretical and functional frameworks for future research in plant lipid biology.

## 1. Introduction

As sessile organisms, plants have developed intricate metabolic and signaling networks to respond to dynamic environmental conditions and coordinate developmental programs. Phospholipid-mediated signaling is a pivotal mechanism for integrating endogenous developmental pathways with external environmental cues. Phospholipids serve as both structural constituents of cellular membranes and signaling molecules that transduce extracellular stimuli into precise intracellular responses. The variability of phospholipid molecules among different plant tissues enables distinct signaling roles, supports tissue-specific developmental processes, and modulates responses to abiotic stresses, including cold, salinity, and hypoxia [1,2,3].

The flexibility of phospholipid biosynthetic pathways enables their structural and functional diversity. Glycerol-3-phosphate (G3P) provides the phospholipid backbone, whereas fatty acids supply acyl chains that undergo highly regulated enzymatic modifications, including elongation, desaturation, and oxidation, to adjust membrane properties. In plants, fatty acids are synthesized in chloroplasts. They are either assembled into glycerolipids within the chloroplast via the prokaryotic pathway or exported to the endoplasmic reticulum for lipid synthesis via the eukaryotic pathway, with some lipids subsequently returning to the chloroplast. This dynamic process facilitates ongoing lipid remodeling [4]. This persistent acyl flux not only coordinates energy homeostasis with membrane functionality but also varies markedly across species and environmental conditions [5,6].

Phospholipids play critical regulatory roles in plant development, often in interaction with phytohormones. Auxin-driven polar localization of PIN-FORMED (PIN) proteins regulates plant morphogenesis. Key enzymes in phospholipid metabolism, including phosphoinositide kinases, phospholipases A/D (PLA/PLD), and cytidine diphosphate-diacylglycerol synthases (CDS), respond to auxin signals. Through these responses, they modulate processes such as axillary shoot branching, vesicular trafficking, and embryogenesis [7,8,9]. PA, a key lipid second messenger, coordinates distinct developmental transitions via direct associations with target proteins. For example, PA directly binds to transcription factors containing AT-hook motifs and to core circadian clock regulators, establishing a metabolite-sensing mechanism that supports feedback regulation of plant development [10,11].

This review synthesizes current insights into phospholipid-mediated regulatory networks in plants, highlighting their contributions to developmental plasticity, stress adaptation, and cell fate specification. We examine conserved signaling pathways across species, highlight emerging themes in lipid signaling, and identify major unresolved questions to guide future research.

## 2. Phospholipid Biosynthesis: Compartmentalization and Key Enzymatic Steps

**Figure 1:**
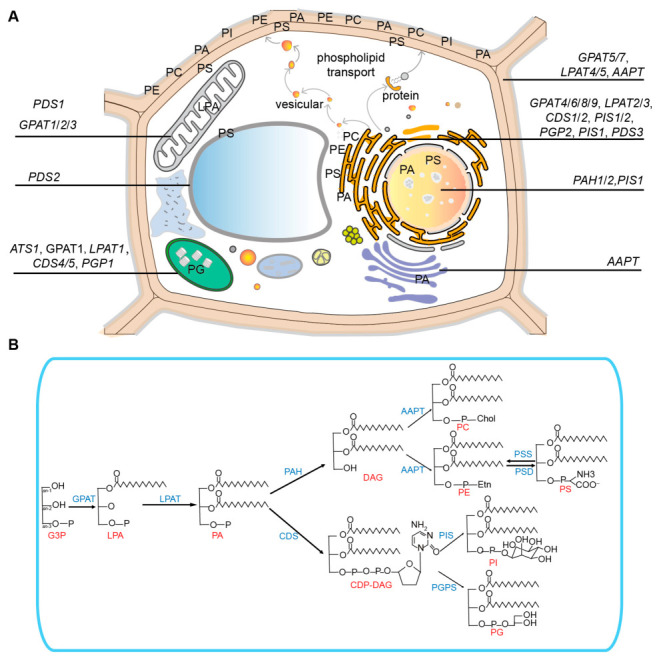
Biosynthesis of phospholipids and subcellular localization of enzymes. (**A**). Subcellular localization of phospholipids and enzymes. Phospholipids are transported to target membranes or other organelles via vesicular trafficking or lipid transfer proteins following synthesis in the ER. The direction of transport is shown by arrows. Abbreviations: G3P, sn-glycerol-3-phosphate; LPA, lysophosphatidic acid; PA, phosphatidic acid; DAG, diacylglycerol; CDP-DAG, cytidine diphosphate diacylglycerol; PC, phosphatidylcholine; PE, phosphatidylethanolamine; PS, phosphatidylserine; PI, phosphatidylinositol; PG, phosphatidylglycerol; GPAT, G3P acyltransferase; LPAT, LPA acyltransferase; PAH, PA phosphatase; AAPT, aminoalcohol aminophosphotransferase; CDS, CDP-DAG synthase; PIS, PI synthase; PGPS, phosphatidylglycerophosphate synthase. (**B**). Proposed pathways for phospholipid head group biosynthesis. The metabolite backbone is shown as a black line, with nodes representing carbon atoms. Side-chain fatty acids are represented by a sixteen-carbon fatty acid (C16). Metabolites are highlighted in red; enzymes are shown in blue.

Phospholipid biosynthesis in plants is a highly compartmentalized process that occurs predominantly within the endoplasmic reticulum (ER) and plastids (Figure 1A). The ER serves as the primary hub for bulk phospholipid production, whereas plastid-localized synthesis is critical for maintaining the thylakoid membrane structure and supporting photosynthetic efficiency [12,13]. G3P acylation initiates the de novo pathway to generate PA, a key metabolic intermediate (Figure 1B). Subsequent phospholipid diversification depends on the incorporation of distinct headgroups into PA, a process catalyzed by compartmentalized enzymes [14]. The substrate specificity of the acyltransferases is governed by the acyl-CoA profile, either plastidial 16:0-CoA or cytosolic 18:1-CoA. This specificity determines the phospholipid composition and reflects a phylogenetic divergence between prokaryotic and eukaryotic pathways [15,16].

### 2.1. G3P Acylation: Functional GPAT and LPAT Diversification

Glycerol-3-phosphate acyltransferases (GPATs) catalyze the initial acylation of G3P at the sn-1 or sn-2 positions (Figure 1B). The ten GPAT homologs in *Arabidopsis* are organized into three functional clades. Plastid-targeted GPATs (ATS1) use acyl carrier protein (ACP) substrates for prokaryotic lipid biosynthesis and contribute approximately 50% of plastidial C16 fatty acids [17,18]. GPAT1 shows high activity toward long-chain and ω-oxidized acyl-CoAs, whereas GPAT2 and GPAT3 show negligible activity toward ω-oxidized substrates [4,19]. GPAT1 is predominantly expressed in flowers and siliques and modulates sn-1 saturation of chloroplast phosphatidylglycerol, whereas GPAT3 is mainly localized in seedlings and leaves [19]. Both GPAT1 and GPAT2 contribute to lysophosphatidic acid (LPA) biosynthesis in mitochondria [20]. Conversely, ER-localized bifunctional GPATs (GPAT4/6/8) acylate G3P via their coordinated acyltransferase and phosphatase activities. Functional redundancy among these isoforms preserves cuticular integrity, as evidenced by the pronounced desiccation phenotype in *gpat4 gpat8* double mutants [21]. Suberin-specific GPATs (GPAT5/7) are selective for very long-chain fatty acids (C16-C24) and have no phosphatase activity. Overexpression of these enzymes leads to increased monoacylglycerol (MAG) accumulation, likely due to the buildup of metabolic intermediates [4,22].

LPATs catalyze acylation at the sn-2 position of LPA, thereby completing PA synthesis (Figure 1B). The plastidial-localized isoform, LPAT1, exhibits a prokaryotic-type preference for 16:0-CoA and is essential for embryogenesis [15]. Under phosphate-limited conditions, ER-localized LPAT2 and LPAT3 regulate flux through the eukaryotic biosynthetic pathway. LPAT2 plays a crucial role in female gametophyte maturation and root elongation [15,23]. Under nitrogen-limiting conditions, LPAT4 and LPAT5 are required for phospholipid and triacylglycerol biosynthesis [24].

### 2.2. PA Channeling: Branchpoints for Membrane Lipid Production and Signaling

PA is directed into two unique enzymatic pathways, leading to distinct metabolic outcomes (Figure 1B). Phosphohydrolases (PAH 1/2) hydrolyze PA to produce DAG, which serves as a precursor for the synthesis of phosphatidylcholine (PC) and phosphatidylethanolamine (PE) [25]. Aminoalcoholphosphotransferase (AAPT) is a bifunctional enzyme that transfers phosphate-choline (P-Chol) and phosphate-ethanolamine (P-Etn) groups to DAG to complete PC and PE formation [26]. Minor phospholipids, including phosphatidylserine (PS), which contains very long-chain fatty acids, are produced through headgroup exchange by PS synthase 1 (PSS1) and are subsequently converted to PE by mitochondrion-targeted PS decarboxylases (PSDs), demonstrating interorganellar lipid trafficking [27,28]. Alternatively, cytidine-diphosphate diacylglycerol synthases (CDSs) convert PA to cytidine diphosphate diacylglycerol (CDP-DAG), a pivotal precursor for acidic phospholipids phosphatidylglycerol (PG) and phosphatidylinositol (PI) (Figure 1B). Tissue-specific CDS isoforms mediate compartmentalized functions: CDS4/5 are critical for preserving thylakoid PG levels, with mutants exhibiting an approximately 40% reduction, whereas CDS1-3 predominantly support PI-dependent signaling pathways [8,29]. Downstream of CDP-DAG, PI synthase (PIS) adds an inositol head group to generate PI [30]. In contrast, phosphatidylglycerophosphate synthase (PGPS) uses CDP-DAG to produce phosphatidylglycerophosphate (PGP) in both plastids and the ER [13,31]. Once synthesized, phospholipids are not static; their acyl chains undergo continuous remodeling that fine-tunes membrane properties and generates signaling intermediates.

## 3. Phospholipid Acyl Remodeling: Enzymatic Networks and Metabolic Flexibility

**Figure 2:**
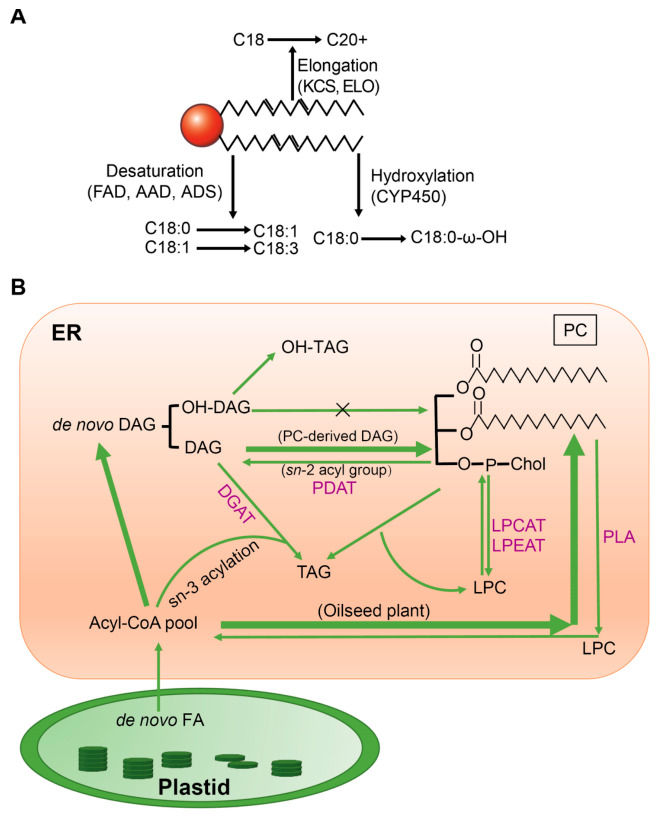
Acyl chain modification and phospholipid remodeling. (**A**): Elongation, desaturation, and hydroxylation of fatty acid chains. C(X:Y) denotes a fatty acid with X carbon atoms and Y double bonds. (**B**): Acyl chain flux in phospholipid biosynthesis, using phosphatidylcholine (PC) as an example. Arrows indicate the direction of acyl chain flux, with arrow thickness representing the relative flux rate. For instance, newly synthesized acyl-CoAs in oilseeds flow predominantly toward LPC. Enzymes are labeled in purple, including acyltransferases and other catalytic enzymes. DAG, diacylglycerol; OH-DAG, hydroxylated diacylglycerol; TAG, triacylglycerol; OH-TAG, hydroxylated triacylglycerol; DGAT, diacylglycerol acyltransferase; PDAT, phosphatidylcholine: diacylglycerol acyltransferase; LPCAT, lysophosphatidylcholine acyltransferase; LPEAT, lysophosphatidylethanolamine acyltransferase.

Acyl remodeling, defined as the post-synthetic alteration or replacement of acyl groups (Figure 2A), is a fundamental mechanism that optimizes membrane biophysical properties and supports phospholipid-dependent signaling in plants [5,32]. Nascent fatty acids (FAs) synthesized in plastids are used for glycolipid and PG biosynthesis. A portion of these FAs is exported to the ER and incorporated into PC, which serves as a primary hub for subsequent acyl remodeling. (Figure 2B) [6,33]. Once esterified into PC, these FAs undergo further elongation, desaturation, or hydroxylation directly on the PC molecule. These modifications alter acyl chain structure and contribute to membrane plasticity. Subsequently, acyl editing generates extensive acyl flux that supports dynamic cellular processes such as membrane fusion and fission [32].

### 3.1. Enzymatic Control of Acyl Chain Architecture

Acyl chain biosynthesis begins with the carboxylation of acetyl-CoA to malonyl-CoA by acetyl-CoA carboxylase (ACCase) within plastids. This is followed by the sequential addition of two-carbon units from malonyl-CoA to ACP-bound acyl chains, generating fatty acids up to C18:0 and C18:1. These acyl chains are subsequently transferred to PC, where they undergo further elongation and desaturation [14]. Within the ER, VLCFAs biosynthesis begins with 3-ketoacyl-CoA synthase (KCS), which catalyzes the condensation of C18-CoA with malonyl-CoA to yield C20 and longer acyl chains. This reaction is the rate-limiting step of the elongation process [34]. Additional enzymes, including ELO family members, further elongate these acyl chains, thereby producing various VLCFAs esterified to PC [35]. Once synthesized, these acyl chains are subject to extensive desaturation.

FA desaturation in *Arabidopsis* is catalyzed by seven acyl-acyl carrier protein desaturase (AAD) isoforms, which supply the C18:1 fatty acid pool [36]. Within this group, *Suppressor of Salicylic Acid Insensitive2* (*SSI2*) was one of the earliest characterized AAD genes; loss-of-function mutations result in a significant accumulation of C18:0 in PC [37]. AAD1/3/4/5 can desaturate C18:0-ACP [25]. Functional specialization among AAD isoforms is reflected in mutant phenotypes: *aad1 aad4* double mutants exhibit a 20% decline in total lipid content, accompanied by a fivefold increase in C16:1 in stems, whereas *aad2 aad3* double mutants display an 85% decrease in ω-7 monoenes in seeds [25].

In addition to AADs, acyl-CoA desaturase-like 1 (ADS) predominantly promotes C26:1 formation in seed lipids, whereas ADS2 is required for the biosynthesis of both C24:1 and C26:1 found in seed lipids, including sphingolipids, PE, and PS [38]. Beyond AAD and ADS desaturases, ER-localized FA desaturases FAD2 and FAD3 cooperatively convert oleate to linolenate. Heterodimer formation between these enzymes promotes metabolic channeling, directly transforming 18:1-PC into 18:3-PC and consequently augmenting polyunsaturated fatty acid (PUFA) biosynthesis [39,40,41]. Concurrently, plastid-localized FAD6/7/8 sustains PUFA biosynthesis within chloroplast lipids [41]. Subcellular relocalization of these desaturases can markedly alter their site specificity and unsaturation patterns [42].

Acyl chain hydroxylation is critical for the biosynthesis of cutin and suberin monomers by providing functional groups for polymerization. Additionally, this modification is also involved in the formation of oxylipin signaling molecules. Cytochrome P450 monooxygenases (CYP450s), notably members of the CYP86 and CYP94 families, catalyze regio- and stereospecific ω-hydroxylation of free FAs and acyl-CoA [43,44], directing them toward polyester assembly or signaling pathways. Acyl oxidation occurs prior to the transfer of acyl chains to glycerol during the production of specialized lipid polymers such as cutin and suberin [4]. Isotope-labeling studies revealed that the hydroxylation of FAs on PC, followed by conversion to DAG, promotes the incorporation of hydroxy-FAs at the sn-2 position of triacylglycerol (TAG). In contrast, hydroxylated DAG does not accumulate efficiently, as it enters an energetically futile cycle of biosynthesis and turnover [6].

### 3.2. Acyl Flux Coordination: Transferases and Hydrolytic Enzymes

Acyl editing refers to the reversible exchange of acyl chains among polar lipid species without changing the net lipid biosynthesis rate. In plants, acyl editing occurs predominantly on PC and limits the accumulation of saturated fatty acids in membrane lipids, thereby modulating plasma membrane fluidity under stress conditions [45,46]. Multiple enzyme families govern this dynamic acyl flux and collectively facilitate lipid remodeling. Lysophospholipid acyltransferases, including lysophosphatidylcholine acyltransferase (LPCAT) and lysophosphatidylethanolamine acyltransferase (LPEAT), catalyze bidirectional acyl exchange at the sn-2 position of PC and PE (Figure 2B). These enzymes usually exhibit a preference for C16:0-CoA substrates [47,48]. Phosphatidylcholine:diacylglycerol acyltransferase (PDAT) catalyzes the transfer of the sn-2 acyl chain from PC to DAG, thereby driving TAG biosynthesis [5,49]. Extensive metabolic labeling studies have shown that the majority of newly synthesized fatty acids are rapidly incorporated into PC through acyl editing pathways in soybean embryos [5], *Brassica* leaves [50], pea leaves [51], and *Arabidopsis* suspension cells [52]. In many cases, this flux surpasses that of the canonical glycerol-3-phosphate acylation pathway. Moreover, PC-mediated acyl editing facilitates fatty acid trafficking and enables preferential channeling of acyl groups without extensive equilibration with the bulk acyl-CoA pool, highlighting its central role in coordinating plant lipid metabolism.

Beyond direct acyl exchange reactions, phospholipases A (PLAs) hydrolyze fatty acids from the sn-1 and sn-2 positions of PC, yielding lyso-PC and free fatty acids (Figure 2B). Subsequently, these products are re-esterified and reintegrated into the acyl editing cycle (Figure 2B). A recent comprehensive review systematically summarizes the catalytic activities and hydrolytic products of phospholipases and critically evaluates their functions in plant growth, development, and responses to environmental stress [53]. The dynamic acyl flux described above has direct implications for plant signaling. One prominent example is the regulation of auxin responses by phospholipid-derived molecules.

## 4. Phospholipid Metabolism as a Regulator for Auxin Dynamics

**Figure 3:**
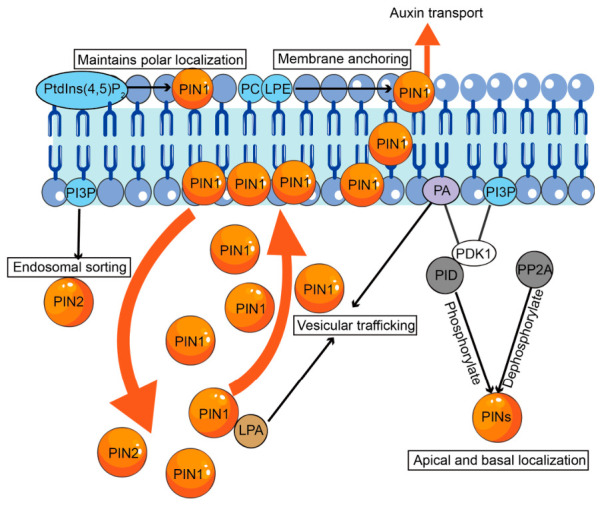
Phospholipid regulation of auxin signaling. PIN1 protein binds to different phospholipids at the plasma membrane and within the cell. Yellow arrows indicate the transport process of PIN1 proteins, while black arrows point from phospholipids to PIN1 proteins. The boxes describe biological reactions involving phospholipid-interacting proteins. Various phospholipids localized on the cell membrane are represented by circles. PI3P, phosphatidylinositol-3-phosphate. PtdIns(4,5)P_2_, phosphatidylinositol-4,5-bisphosphate. PIN, PIN-FORMED. PID, PINOID. PDK1, lipid-related 3-phosphoinositide-dependent protein kinase 1. PP2A, protein phosphatase 2A.

Auxin regulates plant development by controlling polar transport, biosynthesis, and metabolic turnover, thereby establishing asymmetric distribution patterns and concentration gradients essential for growth and environmental adaptation [54]. Phospholipids influence plant developmental processes via extensive crosstalk with auxin signaling pathways (Figure 3) [20,55,56,57].

### 4.1. Phospholipid-Dependent Regulation of PIN1 Trafficking and Localization

PIN proteins are a family of auxin efflux transporters. They mediate directional auxin transport to establish local gradients that are critical for organogenesis, embryonic axis formation, and root meristem patterning [58,59,60,61]. Several phospholipid-mediated signaling pathways interact with PIN proteins, thereby contributing to plant signal transduction and development (Figure 3). PC is essential for stable PIN1 anchoring at the plasma membrane. Methyltransferase XIPOTL1, which is abundant in root transition zones, synthesizes PC and modulates auxin transport. Accordingly, *xiptol1* mutants show reduced PC levels and disrupted PIN1 polarity, leading to impaired root cell elongation [62]. Lysophosphatidylethanolamine (LPE), produced by secretory phospholipase A2 (sPLA2α), enhances PIN1 localization at the plasma membrane by antagonizing endocytic internalization. This was demonstrated by the restoration of PIN1 polarity in *spla2α* mutants following LPE treatment [63]. Furthermore, mitochondrial GPAT-derived LPA directly interacts with PIN1, regulating its vesicular trafficking dynamics to coordinate auxin-driven embryogenesis and seedling morphogenesis [20]. Together, these findings highlight membrane phospholipid as a critical spatial determinant of auxin transport polarity.

### 4.2. PA and PI Derivatives as Lipid Second Messengers in Auxin Signaling Amplification

PA and PI function as critical lipid second messengers that regulate the amplitude and spatial precision of auxin responses. PA, generated by PLDζ2, increases auxin signaling by upregulating early auxin-responsive genes, including *IAA5* and *GH3-3*. Exogenous PA compensates for gravitropic defects in *pldζ2* mutants, underscoring its essential role in auxin-dependent tropic responses [64]. PI modulates auxin signaling by orchestrating endosomal trafficking and protein phosphorylation networks. Specifically, phosphatidylinositol-3-phosphate (PI3P), produced by PI3-kinase (PI3K), directs endosomal transport of PIN2 via SORTING NEXIN1 (SNX1) (Figure 3), a process critical for preserving auxin gradients during root bending [65]. Phosphatidylinositol-4,5-bisphosphate (PtdIns(4,5)P_2_), synthesized by PIP5K1 and PIP5K2, regulates clathrin-mediated endocytosis and maintains polar localization of PIN1, thereby directing auxin distribution and gradient formation essential for embryonic and postembryonic patterning [66,67].

Moreover, 3-phosphoinositide-dependent protein kinase 1 (PDK1) interacts with both PA and PI3P via its pleckstrin homology domain [68,69]. PDK1 promotes the autophosphorylation and activation of PINOID (PID) kinase [70], which then phosphorylates PIN proteins at their central hydrophilic loop (Figure 3). Phosphorylation directs the apical targeting of PINs, whereas dephosphorylation by PP2A enforces their basal localization (Figure 3) [71]. This reversible phosphorylation cycle dynamically regulates PIN-driven auxin transport, establishing a framework for tissue patterning and organogenesis. Collectively, these findings demonstrate that multiple classes of PA and PIPs coordinate distinct steps in PIN trafficking and polarity establishment. This highlights the complexity of phospholipid-mediated regulation of auxin signaling. Beyond its role in auxin signaling, PA functions as a central hub integrating multiple stress and hormone signaling pathways, as elaborated in the following section.

## 5. PA as a Molecular Hub: Binding Partners and Functional Specialization

**Figure 4:**
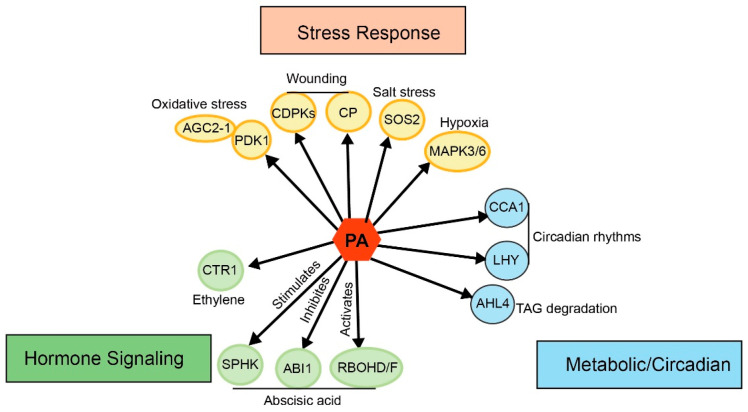
PA-binding proteins regulate plant growth, development, and signal transduction. Black arrows indicate the direction from PA to its interacting proteins. Proteins involved in stress responses are shown in yellow, those involved in hormone signaling in green, and those involved in metabolic reactions and circadian clock regulation in blue. AGC2-1, cAMP-dependent, cGMP-dependent and protein kinase C 2-1. PDK1, phosphoinositide-dependent protein kinase 1. CDPK, calcium-dependent protein kinase; CP, capping protein; SOS2, salt overly sensitive 2; MAPK, mitogen-activated protein kinase; CTR1, constitutive triple response 1; SPHK, sphingosine kinase; ABI1, ABA insensitive 1; RBOHD, respiratory burst oxidase homolog D; CCA1, circadian clock associated 1; LHY, late elongated hypocotyl; AHL4, AT-hook motif nuclear-localized protein 4.

PA, produced via de novo phospholipid synthesis or PLD-mediated hydrolysis, functions as a multifaceted signaling hub that interacts with effector proteins to orchestrate developmental programs and stress-responsive pathways (Figure 4).

### 5.1. Stress-Responsive PA-Protein Networks

Under salt and osmotic stress conditions, PA recruits and activates membrane-associated proteins, orchestrating cellular processes such as ion regulation, endocytic trafficking, and cytoskeletal rearrangements. Salt stress induces PA binding to Salt Overly Sensitive 2 (SOS2), a central kinase in the SOS signaling pathway. This binding increases SOS2 catalytic activity and promotes its localization to the plasma membrane [2]. The SOS2-PA interaction then stimulates Na^+^/H^+^ antiporter SOS1, enhancing Na^+^ efflux and preserving cellular ion balance. PA also directly interacts with mitogen-activated protein kinases 3 and 6 (MAPK3/6), promoting their phosphorylation under hypoxic conditions and integrating MAPK signaling into the stress adaptation network [72]. Kinase cascades are further fine-tuned by PA through PDK1-mediated phosphorylation and activation of the oxidative stress-responsive kinase AGC2-1 [68,69]. During abscisic acid (ABA) signaling, a metabolic feedback loop operates in which cytosolic glyceraldehyde-3-phosphate dehydrogenase (GAPC) stimulates PLDδ activity, coupling glycolytic flux to PA generation [73,74]. Beyond these signaling roles, PA modulates cytoskeletal organization during pathogen infection by activating phosphatidylinositol-4-phosphate 5-kinase and small GTPases. It also suppresses *Arabidopsis* capping protein (CP), which promotes actin filament assembly by inhibiting barbed-end depolymerization. Together, these interactions establish PA as a pivotal signaling intermediate that integrates diverse pathways to coordinate adaptive responses to environmental stress [75,76,77].

### 5.2. Integration of Hormone Signaling

PA functions as a central convergence point for multiple hormone signaling pathways, coordinating developmental and stress-related responses. During ABA-induced stomatal closure, PA generated by PLDα1 directly binds to and suppresses the phosphatase activity of ABI1, a key negative regulator of ABA signaling, thereby strengthening stomatal closure [78]. Sphingosine kinase (SPHK) is also activated by PA, leading to elevated sphingosine-1-phosphate (S1P) production, which further reinforces ABA signal transduction. Distinct PA species, including 16:0-18:2 PA, activate NADPH oxidases RbohD/F, eliciting reactive oxygen species (ROS) bursts necessary for ABA-induced stomatal closure [79]. In ethylene signaling, PA directly interacts with the kinase domain of CONSTITUTIVE TRIPLE RESPONSE 1 (CTR1), weakening its association with ethylene receptors and modulating downstream ethylene-regulated growth and stress responses. Collectively, these findings highlight PA as a multifunctional lipid mediator that integrates hormonal signals across diverse physiological processes [80].

### 5.3. Metabolic and Circadian Coordination

PA also regulates metabolic and circadian processes, extending its role beyond stress and hormone signaling. For example, PA binds to the core circadian transcription factors Late Elongated Hypocotyl (LHY) and Circadian Clock Associated1 (CCA1), impairing their DNA-binding to the promoter of Timing of Cab Expression1 (TOC1) and thereby modulating circadian phase rhythms [11]. Genes encoding PA metabolic enzymes, including PLD, PAH, and Diacylglycerol Kinase 1 (DGK1), display rhythmic expression patterns that couple lipid metabolism to circadian outputs [11]. During seed germination and early seedling growth, PA interacts with the AT-hook transcriptional regulator AHL4, relieving its repression of TAG catabolic genes and promoting lipid mobilization to meet energetic demands [10]. Collectively, these findings indicate that PA integrates lipid metabolism with circadian control and energy homeostasis, enabling adaptive responses at both cellular and organismal levels. While the preceding sections focused on acute signaling responses, phospholipids also exert long-term effects on cell fate determination, a function conserved across kingdoms.

## 6. Phospholipid-Driven Fate Programming in Cells: Conserved Mechanisms Across Kingdoms

**Figure 5:**
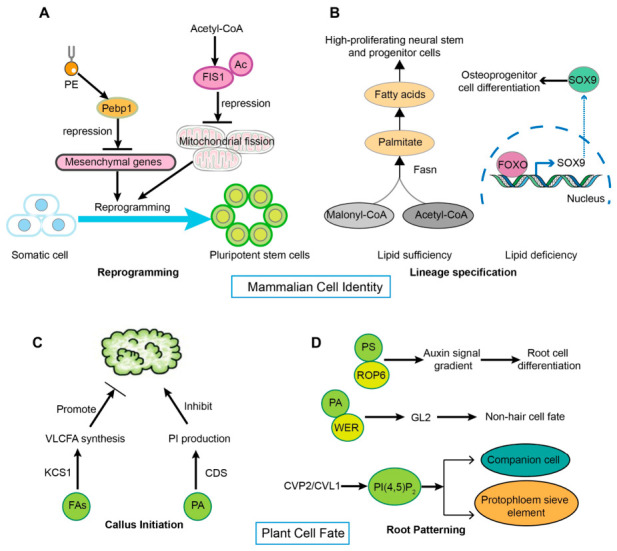
Lipid signaling regulates cell fate reprogramming in animals and plants. (**A**). Lipids influence cell reprogramming. Black arrows indicate the direction from lipids to their interacting proteins. Blue arrows represent the process of somatic cell reprogramming. (**B**). Effects of lipid sufficiency and deficiency on progenitor cells during cell lineage differentiation. Black arrows ultimately point to biological processes, while blue arrows indicate the direction of gene regulation. (**C**). Plant lipid metabolism affects callus formation. Black arrows point from lipids to pluripotent callus, highlighting the role of lipids in plant cell totipotency. VLCFA, very long-chain fatty acid. KCS1, 3-ketoacyl-CoA synthase 1. CDS, cytidine diphosphate-diacylglycerol synthase. (**D**). Phospholipid-binding proteins influence plant cell fate determination. Black arrows indicate the direction from lipids and their interacting proteins to the signaling process, ultimately leading to the establishment of various cell fates. PA, phosphatidic acid. PS, phosphatidylserine. ROP6, Rho-related GTPase from plants 6. WER, WEREWOLF. GL2, GLABRA2.

Phospholipid-based signaling represents an evolutionarily conserved regulatory framework governing cell fate specification in both plant and animal systems (Figure 5). Beyond their structural roles, membrane phospholipids and their metabolic dynamics convey spatial and biochemical cues that inform positional identity and direct developmental decisions. To illustrate this conservation and to highlight mechanistic principles that may inform plant lipid signaling, we first briefly discuss selected examples from mammalian systems.

### 6.1. Lipid Remodeling in Mammalian Cell Identity

Dynamic phospholipid regulation acts as an early determinant of cellular reprogramming by directing metabolic trajectories and influencing fate transitions. In the early stage of mouse cell reprogramming, PE levels rise sharply and help maintain stem cell pluripotency by inhibiting the phosphorylation of downstream IKK kinase [81]. Meanwhile, fatty acid synthesis consumes acetyl-CoA to inhibit acetylation-dependent protein degradation, promoting mitochondrial fission and driving somatic cell reprogramming into induced pluripotent stem cells (Figure 5A) [82]. In differentiated cell lineages, lipid-rich conditions support stem cell proliferation through fatty acid synthase (FASN)-dependent lipogenesis. Conversely, under lipid-limited conditions, activation of FOXO signaling inhibits *SOX9* expression, biasing stem cells toward somatic cell differentiation (Figure 5B) [83,84]. Collectively, these findings highlight lipid metabolism as a pivotal regulatory switch that regulates cell identity decisions in mammalian systems. These mammalian examples underscore the conserved nature of lipid-driven fate programming and reveal key regulatory principles, including metabolic control of cell reprogramming and lipid-mediated protein stabilization, that may also operate in plant systems, as discussed below.

### 6.2. Regulation of Root Cell Fate by Lipid Networks

Cellular totipotency and tissue patterning in plants depend on the spatial regulation of lipid metabolic pathways. During auxin-induced plant regeneration, 3-ketoacyl-CoA synthase 1 (KCS1) restricts pluripotent callus formation from pericycle cells through VLCFA production (Figure 5C) [85]. Disruption of PI biosynthesis in *cds1* and *cds2* mutants impairs callus development, linking plastidial lipid metabolism to cell expansion (Figure 5C) [8]. In the root apical meristem, PS enrichment coincides with auxin flux peaks, whereas PA binding to transcription factor WEREWOLF (WER) represses *GLABRA2* expression, thereby promoting non-hair cell identity (Figure 5D) [86,87]. Moreover, PI(4,5)P_2_ homeostasis is maintained by CVP2/CVL1 and PIP5K-BRX-PAX complexes (Figure 5D). These regulators collectively ensure proper protophloem differentiation and polarized PIN1 localization, thereby contributing to vascular pattern formation [88,89]. These findings demonstrate that lipids function as conserved metabolic cues, integrating developmental signals to direct cell fate decisions across evolutionary lineages.

## 7. Future Perspectives

Despite substantial advances in elucidating phospholipid-mediated signaling in plants, several key questions remain unresolved. First, the molecular mechanisms by which distinct phospholipid species are perceived and translated into specific cellular responses remain poorly understood. The discovery of novel lipid-binding proteins and their downstream effectors, aided by advanced proteomic and lipidomic approaches, will be essential for delineating these signaling cascades. Second, the interplay between phospholipids and other metabolic networks, such as glycolysis, sphingolipid metabolism, and sterol biosynthesis, remains poorly understood and requires systematic investigation to elucidate metabolic feedback during development and stress adaptation. Third, although lipid signaling functions are broadly conserved across kingdoms, the plant-specific diversification and evolutionary trajectories of these components require deeper phylogenetic and functional analyses. Fourth, manipulation of lipid metabolic pathways holds great promise for enhancing crop stress tolerance and productivity. Finally, continued advances in spatial lipidomics and single-cell imaging technologies will facilitate real-time mapping of lipid distributions within defined cells and organelles, providing critical insights into the spatiotemporal regulation of lipid signaling in plants. Addressing these challenges will advance our understanding of lipid biology and accelerate the development of biotechnological applications.

## Data Availability

No new data were created or analyzed in this study. Data sharing is not applicable to this article.
